# Natural product myricetin is a pan-KDM4 inhibitor which with poly lactic-*co*-glycolic acid formulation effectively targets castration-resistant prostate cancer

**DOI:** 10.1186/s12929-022-00812-3

**Published:** 2022-05-09

**Authors:** Jai-Shin Liu, Wei-Kai Fang, Shan-Min Yang, Meng-Chen Wu, Tsan-Jan Chen, Chih-Ming Chen, Tung-Yueh Lin, Kai-Lun Liu, Chien-Ming Wu, Yun-Ching Chen, Chih-Pin Chuu, Ling-Yu Wang, Hsing-Pang Hsieh, Hsing-Jien Kung, Wen-Ching Wang

**Affiliations:** 1 Institute of Molecular and Cellular Biology and Department of Life Sciences, National Tsing-Hua University, Hsinchu, 30013 Taiwan; 2 Department of Biotechnology and Pharmaceutical Technology, Yuanpei University of Medical Technology, Hsinchu, 30015 Taiwan; 3 Institute of Biotechnology and Pharmaceutical Research, National Health Research Institutes, Zhunan, Maioli 35053 Taiwan; 4 Biomedical Translation Research Center, Academia Sinica, Taipei, 11571 Taiwan; 5 Institute of Biomedical Engineering, National Tsing-Hua University, Hsinchu, 30013 Taiwan; 6 Institute of Cellular and System Medicine, National Health Research Institutes, Zhunan, Miaoli 35053 Taiwan; 7 Graduate Institute of Biomedical Sciences, Division of Biochemistry, Molecular and Cellular Biology, Chang Gung University, Taoyuan, 33302 Taiwan; 8 Division of Medical Oncology, Chang Gung Memorial Hospital, Linkou, Taoyuan, 33305 Taiwan; 9 Department of Chemistry, National Tsing Hua University, Hsinchu, 30013 Taiwan; 10 Department of Biochemistry and Molecular Medicine, University of California Davis School of Medicine, University of California Davis Cancer Centre, Sacramento, CA 95817 USA; 11 Graduate Institute of Cancer Biology and Drug Discovery, Taipei Medical University, Taipei, 11031 Taiwan

**Keywords:** Myricetin, Castration-resistant prostate cancer, Histone lysine demethylase family 4 (KDM4), Poly lactic-*co*-glycolic acid (PLGA), Enzalutamide

## Abstract

**Background:**

Castration-resistant prostate cancer (CRPC) with sustained androgen receptor (AR) signaling remains a critical clinical challenge, despite androgen depletion therapy. The Jumonji C-containing histone lysine demethylase family 4 (KDM4) members, KDM4A‒KDM4C, serve as critical coactivators of AR to promote tumor growth in prostate cancer and are candidate therapeutic targets to overcome AR mutations/alterations-mediated resistance in CRPC.

**Methods:**

In this study, using a structure-based approach, we identified a natural product, myricetin, able to block the demethylation of histone 3 lysine 9 trimethylation by KDM4 members and evaluated its effects on CRPC. A structure-based screening was employed to search for a natural product that inhibited KDM4B. Inhibition kinetics of myricetin was determined. The cytotoxic effect of myricetin on various prostate cancer cells was evaluated. The combined effect of myricetin with enzalutamide, a second-generation AR inhibitor toward C4-2B, a CRPC cell line, was assessed. To improve bioavailability, myricetin encapsulated by poly lactic-*co*-glycolic acid (PLGA), the US food and drug administration (FDA)-approved material as drug carriers, was synthesized and its antitumor activity alone or with enzalutamide was evaluated using in vivo C4-2B xenografts.

**Results:**

Myricetin was identified as a potent α-ketoglutarate-type inhibitor that blocks the demethylation activity by KDM4s and significantly reduced the proliferation of both androgen-dependent (LNCaP) and androgen-independent CRPC (CWR22Rv1 and C4-2B). A synergistic cytotoxic effect toward C4-2B was detected for the combination of myricetin and enzalutamide. PLGA-myricetin, enzalutamide, and the combined treatment showed significantly greater antitumor activity than that of the control group in the C4-2B xenograft model. Tumor growth was significantly lower for the combination treatment than for enzalutamide or myricetin treatment alone.

**Conclusions:**

These results suggest that myricetin is a pan-KDM4 inhibitor and exhibited potent cell cytotoxicity toward CRPC cells. Importantly, the combination of PLGA-encapsulated myricetin with enzalutamide is potentially effective for CRPC.

**Supplementary Information:**

The online version contains supplementary material available at 10.1186/s12929-022-00812-3.

## Background

Prostate cancer (PCa) is now the sixth leading cause of cancer-related deaths in men and the first in the UK and second in the United States [1]. Androgen deprivation therapy by surgery or hormonal castration has been the standard treatment for PCa driven by the androgen receptor (AR) pathway since the 1970s [2]. Despite an effective response lasting for a few years, the majority of patients develop a more aggressive form of cancer, referred to as castration-resistant PCa (CRPC). CRPC is primarily stimulated by sustained or even increased AR activity due to AR amplification, AR mutations, and/or the expression of AR splice variants that generate constitutively active forms [3–7] and the upregulation of cytochrome P450 17A1 (CYP17A1), which increases intratumoral androgen synthesis [8]. The second-generation AR antagonist enzalutamide and the inhibitor of CYP17A1, abiraterone, which blocks the synthesis of androgen, have been developed and approved by FDA to improve outcomes for patients with CRPC [8, 9]. While they effectively alleviate symptoms and prolong survival, patients almost always develop resistance, leading to death within a short period. Relapse is common after 1–2 years of treatment and after only 4–6 months in advanced cases (post-chemotherapy) [8, 9]. It is, therefore, necessary to develop a therapeutic modality that can diminish or eliminate aberrantly activated AR activity.

The KDM4 histone lysine demethylase family has recently emerged as a target for tumor therapy [10, 11]. Members of the KDM4/JMJD2 family (KDM4A–KDM4E) catalyze the removal of an *N*ε-methyl group from *N*ε-methyl-lysine residues and belong to dioxygenases with α-ketoglutarate (α-KG) and Fe(II) as cofactors [11, 12]. KDM4A, KDM4B, and KDM4C share a similar domain structure (JmjN, JmjC, two plant homeodomains, and two Tudor domains), while KDM4D and KDM4E lack the *C*-terminal domains (plant homeodomain and Tudor). KDM4A‒KDM4C members act on two histone methyl marks, *i.e.*, histone 3 lysine 9 trimethylation/dimethylation (H3K9me3/me2) and histone 3 lysine 36 trimethylation/dimethylation (H3K36me3/me2) [13, 14], whereas KDM4D and KDM4E only demethylate H3K9me3/me2 [14].

KDM4A–KDM4C enzymes are often overexpressed in several types of malignancies, including prostate, breast, lung, and gastrointestinal tract cancers, lymphoma, and medulloblastoma [10, 15]. Of note, KDM4A and KDM4B interact with AR [16–18]. KDM4A serves as a coactivator of E2F1 [19] and KDM4B regulates AR signaling and turnover to promote tumor progression [17]. KDM4C is upregulated in CRPC [20] and promotes the proliferation of PCa via the activation of c-Myc and AKT [21]. Moreover, KDM4B coactivates c-Jun to regulate the expression of IL-8 [15], enhances autophagy [22], and regulates DNA damage and retrotransposition [23, 24]. By demethylating H3K9me3, a repressive mark enriched in heterochromatin [25, 26], KDM4A–KDM4C function to coactivate AR in the case of PCa, releasing the transcriptional block and promoting tumor progression.

Thus far, two types of KDM4 inhibitors have been reported: α-KG analogues and catalytic-site inhibitors [11]. Of α-KG analogues (*N*-oxalylglycines, hydroxamate analogues, pyridine 2,4-dicarboxylic acids, and 8-hydroxyquinolines), the inhibitor ML324 based on the 8-hydroxyquinoline skeleton shows submicromolar inhibitory activity toward JMJD2E/KDM4E (in vitro), good cell permeability, and in vitro ADME properties [27, 28]. M324 blocks KDM4A-dependent herpes virus-mediated replication in infected cells and displays a promising therapeutic effect by targeting KDM4 [27]. The other 8-hydroxyquinoline -based derivative, B3, blocks the binding of KDM4B to Polo-like kinase 1 to inhibit prostate tumor growth [29]. Recently, potent catalytic-site inhibitors have been developed (Compound 54j and Compound 35) [30, 31]. We have previously identified a catalytic-site inhibitor (NSC636819) that blocks KDM4A/KDM4B-selective inhibition and strongly inhibits LNCaP cell proliferation [18]. However, the in vivo efficacy and other pharmacodynamic features have not been completely characterized.

In this investigation, a natural compound, myricetin, was identified as a pan-KDM4 inhibitor using a structure-guided approach. A poly lactic-*co*-glycolic acid (PLGA)-encapsulated myricetin was developed to enhance its cytotoxicity and bioavailability. We further compared the effects of PLGA-myricetin, enzalutamide, and their combination of tumor growth in vivo.

## Methods

### Data mining of KDM4s expression in UCSC Xena and Oncomine

A comparison of the gene expression of normal and tumor in the prostate cohort in TCGA TARGET GTEx was analyzed by using the UCSC Xena browser (http://xena.ucsc.edu/). mRNA levels of the primary site and metastatic prostate tumor were compared by using the Grasso prostate dataset in Oncomine analysis tools (https://www.oncomine.org/resource/login.html).

### Enzyme assay of KDM4

Formaldehyde dehydrogenase (FDH)-coupled demethylase assay was used to determine the demethylase activity as previously described [18]. All tested compounds were dissolved in dimethyl sulfoxide (DMSO; Sigma-Aldrich, St. Louis, MO, USA) at various concentrations and added to the mixture that the final DMSO concentration is 5%. The reagents for demethylase reactions were dissolved in HEPES (Affymetrix Inc., Cleveland, OH, USA) buffer (50 mM, pH 7.5), except Fe(II) solutions, which were made using (NH_4_)_2_Fe(SO_4_)_2_⋅6H_2_O (Sigma-Aldrich) dissolved in 20 mM HCl (Sigma-Aldrich) to make 800 mM stock solution. All the reagents were stored at -30 ºC. The enzymatic assay was carried out in a final 50 μl reaction mixture containing 50 mM HEPES, pH 7.5, 0.01 U FDH (Sigma-Aldrich), 1 mM NAD^+^ (Sigma-Aldrich), 2 μM of demethylase enzyme, 125 μM ascorbate (Sigma-Aldrich), and 10 μM Fe(II), 5% DMSO, and various concentration of H3K9me3 peptide and α-KG. Each reaction was incubated at 25 ºC for 20 min and the production of NADH was monitored by using the fluorescence spectra (Excitation/Emission: 360/470 nm). All assays were conducted in a 96-well black immune plate (SPL Life Science, Seoul, Korea) and analyzed with a CLARIO star OPTIMA ELISA reader (BMG Labtech., Ortenberg, Germany). The IC_50_ values were obtained by fitting the data to a sigmoid dose–response equation in the GraphPad Prism 7 (GraphPad Software Inc., San Diego, CA, USA).

### Virtual screening

All programs were used in BIOVIA Discovery Studio 2018 (BIOVIA, San Diego, CA, USA). The compounds selected from the database were filtered by Lipinski and Veber Rules, and only used Lipinski Rule of Five [32]. Molecular docking analysis was performed with LibDock [33]. Score Ligand Poses module was used to score the compound’s poses [34], and the docking analysis of scored compounds was performed using CDOCKER [35]. KDM4B was used as a protein model (PDB code: 4LXL). The library containing ~ 60,000 compounds was obtained from the TCM (traditional Chinese medicine) database [36]. Finally, available, virtually optimized candidates were retrieved for the demethylase enzymatic assay.

### Immunoblotting

Total cell lysates were obtained by lysing the cell with buffer [50 mM Tris–HCl pH7.5 (Thermo Fisher Scientific, Waltham, MA, USA), 150 mM NaCl (Honeywell International, Inc., Charlotte, NC, USA), 0.5% Triton X-100 (Sigma-Aldrich), 10% glycerol (Honeywell International, Inc.), 1 mM EDTA (Affymetrix Inc.), and cock-tail protease inhibitors] for 15 min on ice, followed by 10 min of sonication cycle (30 s on, 30 s off) on ice. The level of total histone 3 and trimethylated histone H3K9 was analyzed by western blotting using anti-histone 3 (Cell Signaling, Danvers, MA, USA) and anti-H3K9me3 (Millipore, Burlington, MA, USA).

### MTT assay

Cells (3 × 10^3^) were seeded in a 96-well plate 1 day before the treatment. Cell proliferation was measured by MTT colorimetric assay according to the manufacturer’s instruction (Roche, Indianapolis, IN, USA).

### Drug treatment

Cells were treated with enzalutamide (MDV3100; Active Biochem, Kowloon, Hong Kong, China) and myricetin (Clearsynth, Mumbai, Maharashtra, India), or a combined enzalutamide-myricetin mixture. The IC_50_ value of each drug was determined by treating cells with the drug (0‒160 μM) for three days. For combination evaluation, a twofold serial dilution of each drug and its combination points above and below its IC_50_ value was used in an array format. Combination index (CI) values were calculated by CompuSyn software. The values of the combination dose–response curve, which represents drug-effect-shift analysis, were normalized to IC_50_ values of each single molar concentration of drugs (IC_50_ eq), and the plots were generated by Graphpad Prism 7.

### Preparation of PLGA-encapsulated myricetin

The single-step precipitation method was employed for the preparation of PLGA formulation containing myricetin. Briefly, myricetin and PLGA were dissolved in an oil solution prepared by 62.1 mg of PLGA (Green Square Materials Inc., Taoyuan, Taiwan), 30 mg of D-α-tocopherol polyethylene glycol 1000 succinate (TPGS; Sigma-Aldrich), 3.1 mg cholesterol (Sigma-Aldrich), 3.1 mg of 1,2-dioleoyl-sn-glycero-3-phosphocholine (DOPC; Avanti Polar Lipids, Alabaster, AL, USA), 6.6 mg 1,2-distearoyl-sn-glycero-3-phosphoethanolamine-N-[methoxy-(polyethyleneglycol)-2000] (ammonium salt) (DSPE-PEG2000; Avanti Polar Lipids), and 0.73 mg of myricetin in 1845 μl of DMSO, and added 308 μl oil into the 2150 μl of water dropwise under gentle stirring. The mixture was stirred for 30 min at room temperature, followed by centrifugation at 16,220 rpm to collect the PLGA-myricetin formulation. The PLGA particles were re-suspended by adding 150 μl phosphate saline. The myricetin-loaded and empty formulations were centrifuged at 16,220 rpm for 30 min at 25 °C. The pellet was recovered by DMSO and analyzed by Multiskan SkyHigh Microplate Spectrophotometer (Thermo Fisher Scientific) at 380 nm. The absorbance of the myricetin-loaded formation was normalized by subtracting it with the absorbance of empty particles prepared under the same condition.

### Xenograft

C4-2B cells (1 × 10^6^ cells) were suspended in a culture medium and mixed with Matrigel Matrix (BD, Franklin Lakes, NJ, USA). The mixtures were implanted subcutaneously into six- to eight-week-old male Balb/c nu/nu mice. When the tumor becomes palpable (100 ± 30 mm^3^), PLGA-myricetin (intraperitoneal injected, 20 mg/kg, three times a week), enzalutamide (oral gavage feeding, 12.5 mg/kg, five times a week), and the combined treatment were administered respectively. Tumor volumes were measured for up to 3 weeks. The tumor volume was calculated using the formula: length × width × height × 0.52. Statistical analysis was conducted as described below. The animal protocols for mice experiments were approved by National Tsing Hua University Institutional Animal Care and Use Committee (approval number: NTHU-IACUC-10478) and carried out under the institutional guidelines with animal welfare standards.

### Statistical analysis

Statistical analyses of cell proliferation, quantitative reverse-transcription PCR (qRT-PCR), and enzymatic experiments were performed by student’s *t*-test using the GraphPad Prism 7. The statistical analyses of xenograft analysis were performed by student’s *t*-test using the GraphPad Prism 7. A *p-value* < 0.05 was considered statistically significant.

## Results

### KDM4A, KDM4B, and KDM4C are overexpressed in PCa

**Fig. 1:**
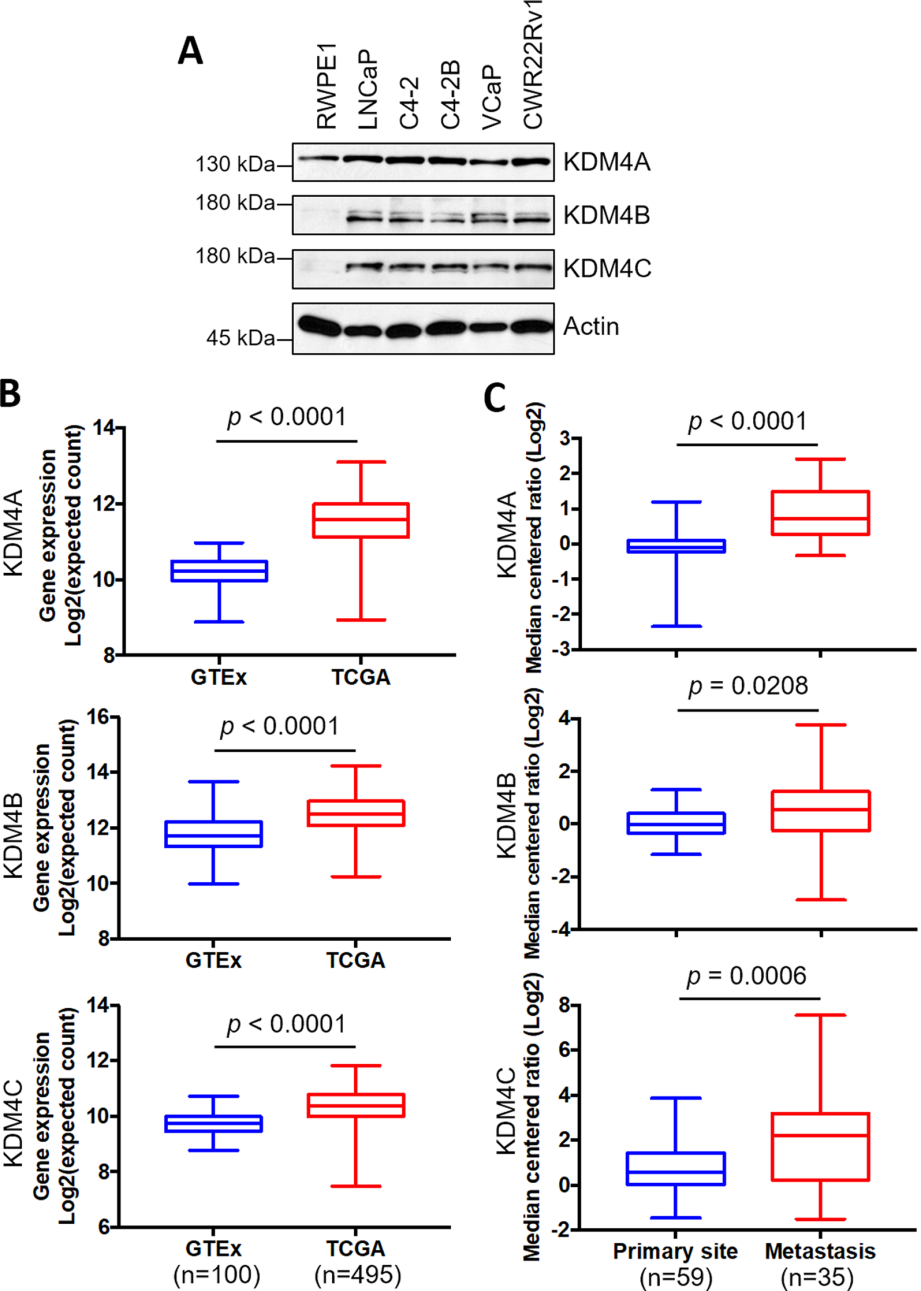
KDM4A, KDM4B, and KDM4C are overexpressed in PCa. **A** Expression of KDM4A, KDM4B, and KDM4C in an immortalized prostatic epithelial cell line (RWPE1) and PCa cell lines (LNCaP, C4-2, C4-2B, VCaP, and CWR22Rv1) using Western blotting analysis. Actin served as an internal control. **B** Expression of *KDM4A*, *KDM4B*, and *KDM4C* in PCa (TCGA) and normal (GTEx) specimens based on the UCSC Xena tool (https://xenabrowser.net). **C** Analysis of expression of KDM4A, KDM4B, and KDM4C in primary and metastatic PCa tissues based on the Grasso prostate dataset in Oncomine database (https://www.oncomine.org/resource/login.html). Statistical significance was determined using Student's *t*-test

We first evaluated the expression of KDM4A, KDMB, and KDM4C in several prostatic epithelial lines: the immortalized prostatic epithelial line RWPE1, the androgen-dependent PCa line LNCaP, and the AR-positive CRPC lines C4-2, C4-2B, VCaP, and CWR22Rv1. Figure 1A shows that the levels of KDM4A, KDM4B, and KDM4C were much higher in cancer cell lines than in RWPE1. We further evaluated the expression level of KDM4s in PCa samples (TCGA, n = 495) and normal samples (GTEx, n = 100) using the online Xena tool (https://xenabrowser.net). As shown in Fig. 1B, levels of *KDM4A*, *KDM4B*, and *KDM4C* were significantly higher in PCa tumors than in normal tissues. Additionally, *KDM4A*, *KDM4B*, and *KDM4C* were significantly higher in the metastatic tumor specimens than in primary tissues based on the Grasso prostate dataset in the Oncomine database (https://www.oncomine.org/resource/login.html) (Fig. 1C). These results together suggest that KDM4A/KDM4B/KDM4C are overexpressed in PCa tumors, particularly in metastatic tumors.

### Myricetin inhibits KDM4A, KDM4B, and KDM4C, as revealed by a structure-based screening from a natural compound library

Accumulating evidence shows natural products displaying anti-PCa activities [37]. We have thus searched for putative KDM4 inhibitors in the Traditional Chinese Medicine Database@Taiwan [36] guided by a structural-based approach using the KDM4B structure. Compounds (n = 20,781) were selected from the database (n = 60,563) based on Lipinski rules of five [32]. LibDock docking program in BIOVIA Discovery Studio [33] was carried out to derive drug-like hits (n = 7565; LibDock score greater than 120). An additional scoring using the program of Score Ligand Poses [34] obtained 322 hits with LigScore1 and LigScore2 greater than 0. We then carried out docking analysis with CDOCKER [35] to derive the best 23 virtual hits (–CDOCKER Energy greater than 30 kcal/mol and –CDOCKER Interaction Energy greater than 40 kcal/mol) (Additional file 1: Fig. S1). These compounds were subjected to in vitro KDM4B demethylase assay.

Enzymatic analysis of available compounds revealed that three flavonoid compounds (quercetin, kaempferol, and myricetin) exhibited potent inhibitory effects, particularly myricetin and quercetin (relative activity: 5.3% for quercetin, 2.9% for myricetin; IC_50_: 2.40 μM for quercetin, 1.30 μM for myricetin) (Additional file 1: Table S1). Myricetin, the most potent one, also displayed inhibitory activity toward KDM4A and KDM4C (IC_50_: 1.13 μM for KDM4A, and 1.12 μM for KDM4C), indicating a pan-KDM4 inhibitor.

**Fig. 2:**
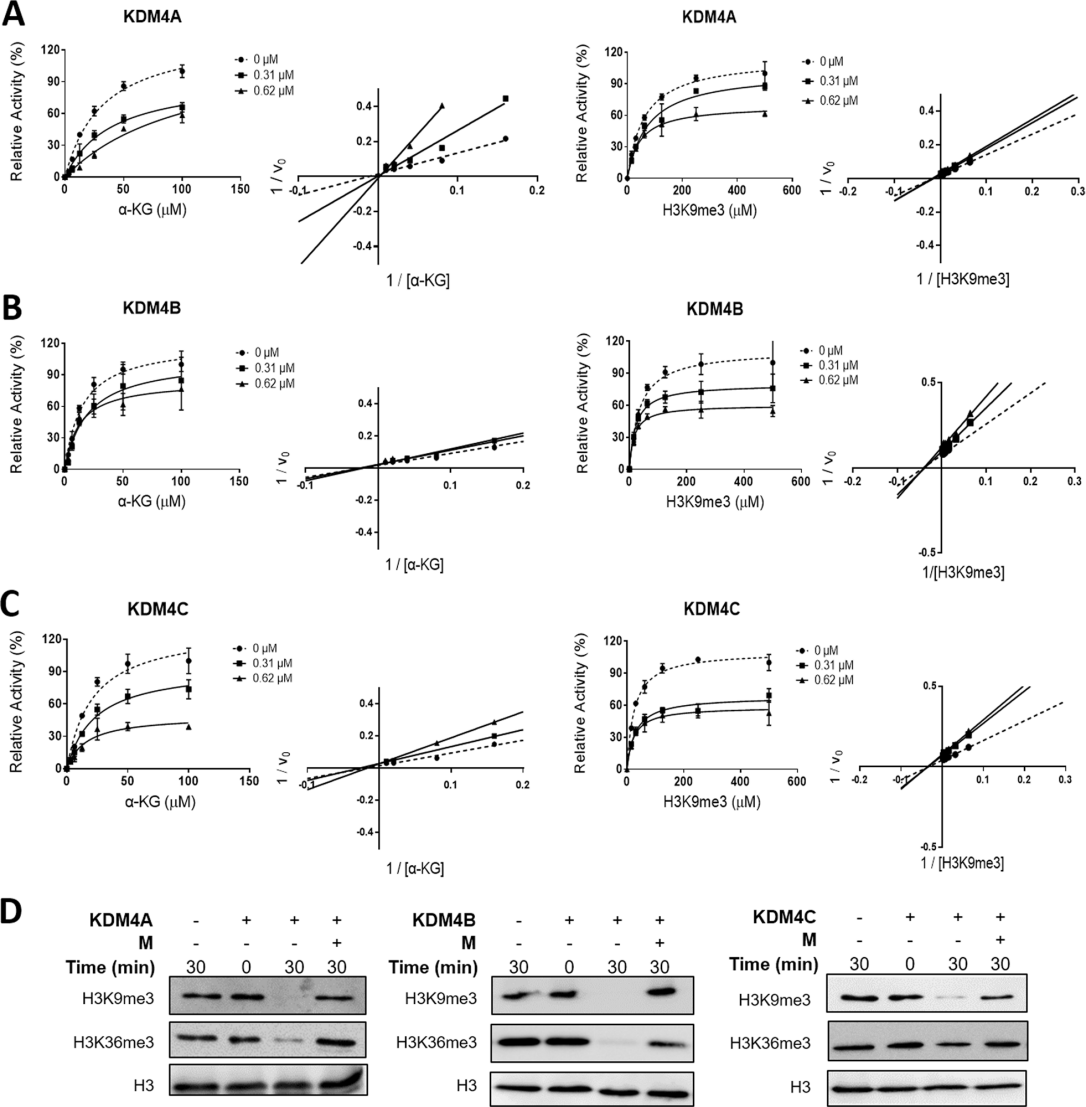
Myricetin inhibits the demethylation activity of KDM4A, KDM4B, and KDM4C. **A**–**C** Kinetic analyses of myricetin that inhibited the demethylation activity of KDM4A (**A**), KDM4B (**B**), and KDM4C **C** with increasing concentrations of α-KG (kinetics, the first raw; and the corresponding Lineweaver–Burk plot, the second raw), or with increasing concentrations of the H3K9me3 peptide (kinetics, the third raw; and the corresponding Lineweaver–Burk plot, the fourth raw). **D** Inhibition of H3 demethylation by KDM4A, KDM4B, or KDM4C in the presence of 50 μM myricetin using Western blotting analysis. The reaction mixture containing 10 μM enzyme, 100 μM inhibitor or blank buffer, and 5 μM of H3 in 50 mM HEPES, pH 7.5, 1 mM αKG, 2 mM ascorbate, and 50 μM Fe(II) was incubated at 37 °C for 30 min, followed by western blot analysis. H3 lysine modifications were probed with H3K9me3 and H3K36me3 antisera, respectively. M, myricetin

We next determined the inhibitory kinetics of myricetin on KDM4A − C. With increasing concentrations of α-KG, myricetin inhibited the demethylation activity of KDM4A, KDM4B, and KDM4C in a competitive manner (Fig. 2A–C). A Lineweaver–Burk plot (Fig. 2A–C) demonstrated myricetin competes with α-KG for binding to the active site (*K*_*i*_ = 0.21 μM for KDM4A, 0.53 μM for KDM4B, and 0.15 μM for KDM4C). Kinetic analysis in the presence of increasing concentrations of the H3K9me3 peptide revealed that myricetin blocked the demethylation activity of KDM4A, KDM4B, and KDM4C by a non-competitive mode (Fig. 2A − C; *K*_*i*_ = 1.26 μM for KDM4A, 1.01 μM for KDM4B, and 0.62 μM for KDM4C), indicating that myricetin did not bind to the H3K9me3 peptide-binding region (Fig. 2). Using histones as the substrate, myricetin blocked the demethylation of H3K9me3 or H3K36me3 by KDM4A, KDM4B, or KDM4C (Fig. 2D).

We next performed molecular docking to build a KDM4B⋅myricetin complex model using BIOVIA, Discovery Studio (CDOCKER) since we were not able to obtain well diffracting myricetin-liganded KDM4 crystals. As shown in Additional file 1: Fig. S2, myricetin occupied the catalytic pocket of KDM4B through extensive H bonds (Asn87, Gly171, Ala187, Asn199, Lys242, Ser289, and Asn291). These seven H bonds form a strong network between myricetin and KDM4B, which makes myricetin a potent inhibitor (Additional file 1: Fig. S2A − B). The 3-hydroxy benzene moiety occupied the α-KG site of KDM4B with four H bonds [18], and the 4H-chromen-4-one moiety of myricetin occupied the other extensive residues in the catalytic pocket of KDM4B with three H bonds and other strong non-bond interactions like Pi-anion (Asp136 and His189) and Pi-Pi stacked interactions (His189) (Additional file 1: Fig. S2A, B).

We further evaluated the inhibitory effects of analogues based on three separate parts of myricetin: (4-phenylpiperazine-1-yl) (phenyl) methanone, (E)-N'-(benzylidene) isonicotinohydrazide, and pyrocatechol (Additional file 1: Tables S2, S3, and S4). Among those, BPRKD63S0 (3,4-dihydroxybenzoic acid; 63S0) which belongs to an analogue of the pyrocatechol exhibited the highest inhibitory effect (560 nM). In addition, we have determined the crystal structure of the KDM4A⋅63S0 complex at 2.6 Å (Additional file 1: Table S5; PDB code: 7EQV). The inhibitor 63S0 coordinates the metal ion with two hydroxyl groups and makes two H bonds with Tyr132, Lys206 (Additional file 1: Fig. S3). Superposition of the KDM4A⋅63S0 crystal structure and the KDM4B⋅ myricetin model revealed the same β-jellyroll structural fold (Additional file 1: Fig. S4A). Additionally, 63S0 and the pyrocatechol moiety of myricetin share overlapped surrounding residues in the α-KG-binding site (Additional file 1: Fig. S4B), consistent with a highly conserved active-site framework seen for the structures of KDM4A‒C [18]. These results collectively suggest myricetin interacts with the metal-binding site and serves as a potent α-KG-type inhibitor that blocks KDM4s.

### Myricetin effectively inhibits the growth of C4-2B and blocks H3K9me3 demethylation

**Fig. 3:**
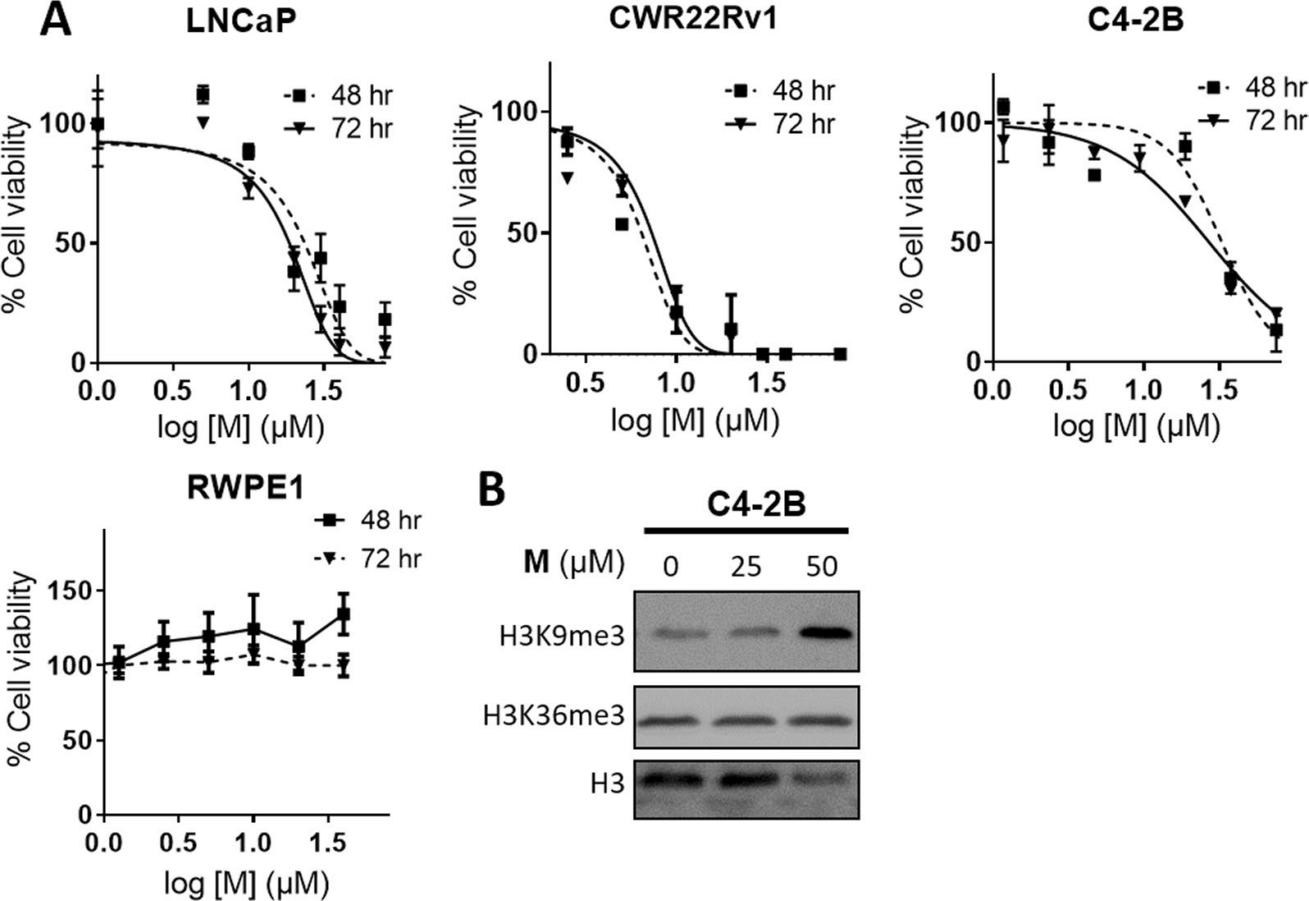
Myricetin exhibits cell cytotoxicity against androgen-dependent and -independent PCa cells. **A** Cells (normal immortalized RWPE-1, androgen-dependent LNCaP, and androgen-independent C4-2B, CWR22Rv1) were treated with different concentrations of myricetin over 3 days as indicated, followed by MTT assay. **B** H3K9me3 and H3K36me3 levels in myricetin-treated C4-2B cells for 2 days. The H3K9me3, H3K36me3, and H3 signals were detected in cell lysates by western blot analysis. *M* myricetin

We evaluated the effect of myricetin on the growth of AR-positive cells. As shown in Fig. 3A, cell viability was significantly reduced in myricetin-treated LNCaP cells after 3 days of culture (IC_50_ = 18.5 ± 0.8 μM). The proliferation of the CRPC cell line C4-2B was also blocked by myricetin (IC_50_ = 20.6 ± 0.9 μM). In contrast, myricetin had essentially no effect on the immortalized normal RWPE-1 line. A higher level of H3K9me3 was observed in C4-2B cells treated with myricetin than in untreated cells, while a comparable H3K36me3 signal was observed, suggesting that myricetin specifically blocked H3K9me3 demethylation in C4-2B cells and reduced cell proliferation (Fig. 3B). These results provide strong evidence that myricetin is a potent natural inhibitor of AR-positive PCa cells (LNCaP and CRPC line C4-2B).

**Fig. 4:**
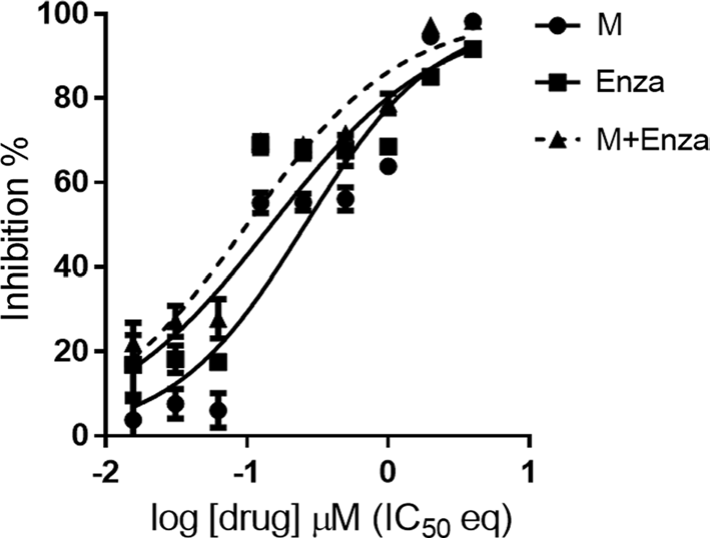
Effects of myricetin and enzalutamide alone and in combination. C4-2B cells were treated with indicated concentrations of the drugs (M, myricetin; Enza, enzalutamide; M + Enza: the combination of myricetin and enzalutamide). The ratio of Myricetin/enzalutamide is [IC_50_]_Myricetin_/[IC_50_]_enzalutamide_ based on the Chou-Talalay method [39]. Cell viability was measured using the MTT assay, the non-linear regression trendline analysis of IC_50_ values was calculated by Prism7 and the CI values were generated by CompuSyn

Enzalutamide is a second-generation AR inhibitor for the treatment of metastatic CRPC [38]. Accordingly, we evaluated the effects of combined treatment of enzalutamide with myricetin using the Chou-Talalay method [39]. In particular, we tested the sensitivity of C4-2B using the constant ratio combination design [39]. The combination index (CI) was calculated for the evaluation of synergism (where CI < 1 indicated a synergistic effect) [39]. As shown in Fig. 4, the combined drug treatment had a greater inhibitory effect than those of either treatment alone, with low CI values (0.72 ± 0.22), suggesting that the drugs have synergistic effects.

### PLGA-encapsulated myricetin formulation plus enzalutamide is more effective than enzalutamide alone

**Fig. 5:**
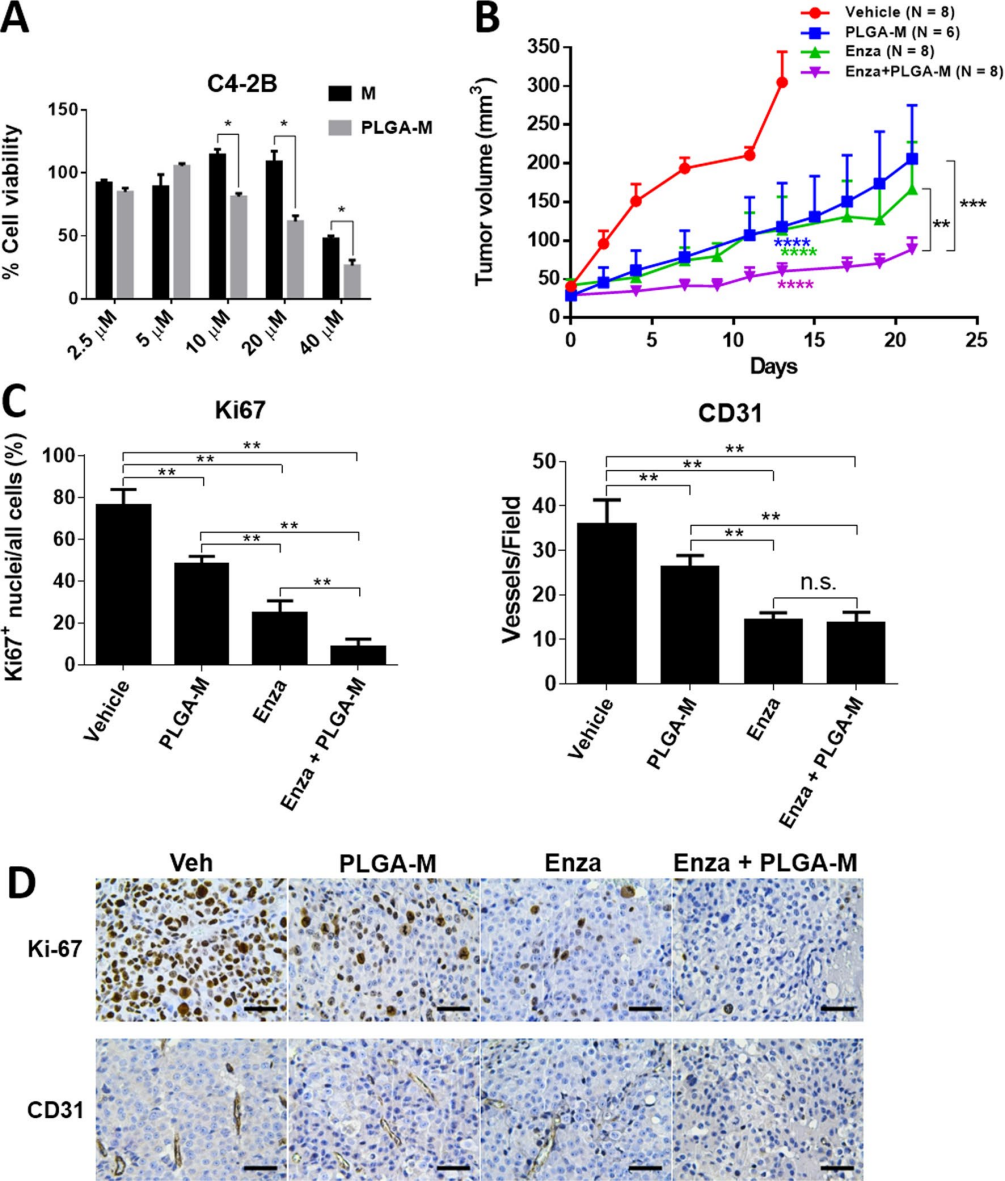
Myricetin impairs tumor growth in C4-2B xenografts. **A** Comparison of cell viability of C4-2B treated with free-form myricetin (Free M) or PLGA-encapsulated myricetin (PLGA-M). Cells were treated with different concentrations of M or PLGA-M for 24 h, followed by MTT assay. **B** C4-2B cells (1 × 10^6^) were implanted subcutaneously in the hindlimb of nude mice. PLGA-myricetin formulation (PLGA-M) (intraperitoneal injected, 20 mg/Kg, three times a week), enzalutamide (Enza) (oral gavage feeding, 12.5 mg/Kg, five times a week), and the combined treatment (Enza + PLGA-M) were administered respectively after 14 days implantation. Tumor volumes were measured for up to 3 weeks. Tumor volume was calculated using the formula (length × width × height × 0.52). Statistical significance was evaluated using Student’s *t*-test. **C** Representative IHC images of Ki67 and CD31 analysis of xenograft tumor sections from mice treated with vehicle, PLGA-M, Enza, or Enza + PLGA-M. **D** The proportion of Ki67 + cell was rescored in each field (400 ×) for six random fields in each group of IHC images. CD31 positive vessels in each field (100 ×) were scored for six random fields in each group. Significance was calculated using Student’s *t*-test. *P < 0.05; **P < 0.01; ***P < 0.001

Although myricetin has promising beneficial effects in human cell culture, animal models, and human clinical studies [40], it is poorly absorbed in vivo, possibly owing to its short half-life in plasma [41–43]. To increase its bioavailability, a polymer-entrapped formulation of myricetin was generated using the FDA-approved drug carrier PLGA which effectively prolongs drug blood circulation, gains enhanced permeability and retention effect for IV injection, and serves as a good platform to conjugate tumor-targeting ligands [44, 45]. The encapsulation efficiency of myricetin in the PLGA formulation was 55%. The PLGA‒myricetin displayed greater cytotoxicity than that of free myricetin at 10 − 40 μM (Fig. 5A).

To assess the efficacy of PLGA‒myricetin in vivo, xenografts generated from C4-2B cells were treated with vehicle, PLGA‒myricetin, enzalutamide, and the combination of PLGA‒myricetin and enzalutamide. As compared with the vehicle, treatment with PLGA‒myricetin (*p* = 0.0002), enzalutamide (*p* = 0.0002), or the combination of PLGA‒myricetin and enzalutamide (*p* = 0.0002), led to a significant impairment of tumor growth in xenografts at day 13 (Fig. 5B). Significantly, a lower level of tumor volume was detected in the combined treatment than in the PLGA‒myricetin treatment (*p* = 0.0002) and in the enzalutamide treatment (*p* = 0.0002) at day 21 (Fig. 5B).

Expression of the proliferation marker Ki-67 was significantly lower in PLGA-myricetin-treated xenografts and enzalutamide-treated xenografts than in the vehicle group (Fig. 5C). The combined treatment, remarkably, exhibited essentially no positive Ki-67 signals (Fig. 5D). We also examined the endothelial cell-specific marker CD31 in xenografts. A significantly higher level of CD31 was detected in the vehicle group than in the PLGA-myricetin-treated and enzalutamide-treated groups (Fig. 5C). Little CD31 was detected in the combined treatment group. These results suggest that PLGA–myricetin effectively blocked the proliferation in C4-2B and xenograft models. Furthermore, the rate of tumor growth was lower for the combined myricetin–enzalutamide treatment than for enzalutamide alone.

## Discussion

Accumulating evidence supports the oncogenic potential of KDM4A, KDM4B, and KDM4C, which are overexpressed in several types of cancer, including PCa [10, 11]. Analyses of KDM4B silencing using a lentivirus-based approach or small-drug inhibitors suggest that KDM4B is a potential therapeutic target against androgen-sensitive PCa [18]. Additionally, KDM4B co-activates c-Myc to directly promote c-Myc-mediated tumor metabolism contributing to CRPC progression [46]. Targeting KDM4B thus represents a potential strategy for CRPC driven by c-Myc and AR. In this study, we identified a natural compound, myricetin, capable of blocking KDM4B as well as KDM4A and KDM4C demethylation activity (H3K9me3 and H3K36me3) using a structure-guided approach. We showed that myricetin reduced the growth of androgen-sensitive LNCaP cells as well as androgen-resistant, AR-positive CRPC cell models (CWR22Rv1 and C4-2B), while it did not affect immortalized prostate epithelial cells. Our findings were consistent with the finding that KDM4B downregulation in KDM4B-knockdown C4-2B cells impaired tumor growth in C4-2B xenografts [46]. Of note, KDM4A knockdown or KDM4C knockout in castration-resistant PCa cells also leads to decreased tumor formation [19, 21]. Interestingly, KDM4A, KDM4B, and KDM4C, which share a relatively highly conserved JmjC domain and activity, display overlapping, but distinct spatiotemporal expression profiles. As such, their functions are not completely redundant. They have distinct sets of target genes and work in concert, in a combinatorial and complementary way [47, 48]. Given that both possess oncogenic potential, the development of a pan-KDM4 inhibitor is a promising strategy for advanced PCa.

Three flavonoid compounds (quercetin, kaempferol, and myricetin) displayed the strongest inhibitory effects, particularly myricetin and quercetin (IC_50_: 1.30 μM for myricetin and 2.40 μM for quercetin). A structural difference is noted in the pyrocatechol part of these compounds: myricetin with three -OH groups, quercetin with two, and kaempferol with one. Docking analysis of KDM4B⋅myricetin reveals higher interaction energy (Additional file 1: Fig. S2). We have thus chosen myricetin for the subsequent analysis. We showed that myricetin exhibits competitive inhibitory activity against α-KG and non-competitive inhibitory activity against the H3K9me3 peptide. A docking simulation revealed that the pyrogallol moiety from myricetin interacts with Ser289 via an H bond and makes Pi-Pi interactions with His189 at the α-KG-binding site, whereas the chromen-4-one moiety makes H bonds with Asn87, Asp136, Gly171, and Lys242 at the peptide-binding region (Additional file 1: Fig. S2) [18, 49]. Additionally, we have determined the KDM4A⋅63S0 complex structure in which 63S0 is a pyrocatechol analogue. Of note, 63S0 coordinates the metal ion and makes H bonds with Tyr132, Lys206 in the α-KG-binding region of the catalytic pocket. These results collectively suggest myricetin is bound at the center of the active site and functions as a potent α-KG-type inhibitor that blocks the demethylation activity by KDM4s. Importantly, myricetin significantly decreased the viability of AR-positive LNCaP and C4-2B cells. Furthermore, it inhibited histone demethylase activity on H3K9me3 by KDM4A − C in C4-2B cells, suggesting that myricetin is a potent natural compound against demethylation by KDM4A − C in C4-2B cells.

Myricetin, by its nutraceuticals value, has drawn much attention to understanding its molecular mechanisms for medicinal translation. We have shown that myricetin effectively inhibits the growth of prostate cancer lines LNCaP, C4-2B, and CWR22rv1 but not the immortalized normal RWPE-1. Importantly, we have shown that myricetin which targets KDM4A − C curbs tumor growth in C4-2B xenografts, suggesting an anti-cancer agent in prostate cancer. Chaudhary et al*.* recently report that flavonoids including myricetin inhibit the enzymatic activity of cytochrome P450 enzymes (CYP1A1 and CYP1B1) [50]. Interestingly, Chaudhary et al*.* show that myricetin exhibits the highest inhibitory effect using the 2,3,7,8-tetrachlorodibenzo-*p*-dioxin-induced CWR22rv1 cells which constitutively expresses CYP1 enzymes. It is thus possible that myricetin also targets CYP1 enzymes to display an anti-carcinogenic effect, which adds a chemoprotective role of myricetin in prostate cancer prevention. Further investigation is required to clarify the biological targets of myricetin and its underlying mechanisms involved in anti-carcinogenic/anti-proliferative activity in prostate cancer.

Despite the potential health benefits of myricetin including anti-oxidative, anti-inflammatory, anti-cancer, and antidiabetic activities [51], its poor bioavailability through the oral route limits its pharmaceutical application [43]. To overcome this issue, we developed a PLGA-based vehicle for the delivery of myricetin. Notably, PLGA, with its excellent biocompatible and biodegradable features, has been successfully utilized to entrap leuprorelin acetate, an agonist analogue of gonadotropin-releasing hormone, as a lipophilic synthetic polymer microsphere, allowing prolonged release for the treatment of advanced PCa [52–54]. The PLGA–myricetin formulation, interestingly, displayed a lower IC_50_ value toward C4-2B than that of free myricetin, indicating that this form might enhance cellular uptake or penetration [44]. Remarkably, PLGA-myricetin significantly reduced the tumor volume in C4-2B xenografts compared with that in the vehicle group. The combined administration of enzalutamide and myricetin essentially blocked tumor growth in C4-2B xenografts. Furthermore, an in vitro efficacy analysis showed that myricetin and enzalutamide have synergistic effects. Thus, targeting the co-activator KDM4 has the potential to control the growth of CRPC driven by the AR pathway. Further investigations are needed to optimize the formulation to increase the synergistic effect in vivo.

## Conclusions

In summary, a natural compound, myricetin, was identified as a pan-KDM4 inhibitor able to block demethylation activity (H3K9me3 and H3K36me3). Myricetin exhibited potent cell cytotoxicity toward LNCaP as well as CRPC cells (C4-2B and CWR22Rv1) but not toward normal immortalized cells. Importantly, we provide clear evidence that myricetin encapsulated in PLGA alone or in combination with enzalutamide effectively curbs tumor growth in C4-2B xenografts. Our results highlight a potential intervention strategy for CRPC.

## Supplementary Information

**Additional file 1**: **Fig. S1.** Schematic diagram of KDM4B inhibitor discovery from a library of natural products. **Fig. S2.** Analysis of interaction residues in the KDM4B-compound models. (A) The docked KDM4B·myricetin complex. Myricetin, M, occupied the catalytic pocket of KDM4B. (B) 2D diagram of KDM4B·myricetin interaction. Myricetin has seven H bonds, two Pi-anion, one Pi-Pi stacked, one Pi-Pi T-shaped, and one interaction with KDM4B. **Fig. S3.** The $2{F}_{o}-{F}_{c}$ map of 63S0 in the liganded complex, contoured at 1.5 σ. The bound 63S0 is drawn as heavy blue sticks. The interacting residues are drawn in thin grey sticks. The oxygen and nitrogen atoms are colored in red and blue, respectively. The Nickel ion is drawn in green. **Fig. S4.** Superposition of KDM4A and KDM4B structures. (A) The KDM4A⋅63S0 crystal structure (orange) and the modeled KDM4B⋅myricetin structure (slate blue) are superimposed. (B) Superposition of active-site residues based on (A). The bound 63S0 and the nearby residues are shown as thin sticks. Myricetin and the surrounding residues are shown as thick sticks. The oxygen and nitrogen atoms are red and blue, respectively. **Table S1.** Inhibition effect of selected compounds from TCM database on KDM4B. **Table S2.** Inhibition effects of analogues based on a myricetin’s fragment, (4-phenylpiperazine-1-yl) (phenyl) methanone, on KDM4A. **Table S3.** Inhibition effects of analogues based on a myricetin’s fragment, (E)-N'-(benzylidene) isonicotinohydrazide, on KDM4A. **Table S4.** Inhibition effects of analogues based on a myricetin’s fragment, pyrocatechol, on KDM4A. **Table S5.** Crystallographic data and refinement statistics.

## Data Availability

The data presented in the study are included in the article and supplementary information.

## Abbreviations

ADME: Absorption, distribution, metabolism, and excretion
AR: Androgen receptor
CRPC: Castration-resistant prostate cancer
PCa: Prostate cancer
PLGA: Poly lactic-*co*-glycolic acid
α-KG: α-Ketoglutarate
KDM4: Histone lysine demethylase family 4
CI: Combination index
DMSO: Dimethyl sulfoxide
CYP: Cytochrome P450
H3K9me3/me2: Histone 3 lysine 9 trimethylation/demethylation
H3K36me3/me2: Histone 3 lysine 36 trimethylation/demethylation
qRT-PCR: Quantitative reverse-transcription PCR
TPGS: D-α-tocopherol polyethylene glycol 1000 succinate
DOPC: 1,2-Dioleoyl-sn-glycero-3-phosphocholine
DSPE-PEG2000: 1,2-Distearoyl-sn-glycero-3-phosphoethanolamine-N-[methoxy-(polyethyleneglycol)-2000] (ammonium salt)
