# Supplementary material for: Natural product myricetin is a pan-KDM4 inhibitor which with poly lactic-co-glycolic acid formulation effectively targets castration-resistant prostate cancer
Source: J Biomed Sci. 2022 May 9;29:29. doi: 10.1186/s12929-022-00812-3 (PMC9082844; doi:10.1186/s12929-022-00812-3)
Supplement: Supplementary file 1 — Additional file 1: Fig. S1. Schematic diagram of KDM4B inhibitor discovery from a library of natural products. Fig. S2. Analysis of interaction residues in the KDM4B-compound models. (A) The docked KDM4B·myricetin complex. Myricetin, M, occupied the catalytic pocket of KDM4B. (B) 2D diagram of KDM4B·myricetin interaction. Myricetin has seven H bonds, two Pi-anion, one Pi-Pi stacked, one Pi-Pi T-shaped, and one interaction with KDM4B. Fig. S3. The $2{F}_{o}-{F}_{c}$2Fo-Fc map of 63S0 in the liganded complex, contoured at 1.5 σ. The bound 63S0 is drawn as heavy blue sticks. The interacting residues are drawn in thin grey sticks. The oxygen and nitrogen atoms are colored in red and blue, respectively. The Nickel ion is drawn in green. Fig. S4. Superposition of KDM4A and KDM4B structures. (A) The KDM4A⋅63S0 crystal structure (orange) and the modeled KDM4B⋅myricetin structure (slate blue) are superimposed. (B) Superposition of active-site residues based on (A). The bound 63S0 and the nearby residues are shown as thin sticks. Myricetin and the surrounding residues are shown as thick sticks. The oxygen and nitrogen atoms are red and blue, respectively. Table S1. Inhibition effect of selected compounds from TCM database on KDM4B. Table S2. Inhibition effects of analogues based on a myricetin’s fragment, (4-phenylpiperazine-1-yl) (phenyl) methanone, on KDM4A. Table S3. Inhibition effects of analogues based on a myricetin’s fragment, (E)-N'-(benzylidene) isonicotinohydrazide, on KDM4A. Table S4. Inhibition effects of analogues based on a myricetin’s fragment, pyrocatechol, on KDM4A. Table S5. Crystallographic data and refinement statistics. [file 12929_2022_812_MOESM1_ESM.docx]

**Additional file 1**

**Natural product myricetin is a pan-KDM4 inhibitor which with poly lactic-*co*-glycolic acid formulation effectively targets castration-resistant prostate cancer**

Jai-Shin Liu^1,2†^, Wei-Kai Fang^1,3†^, Shan-Min Yang^1^, Meng-Chen Wu^1^, Tsan-Jan Chen^1^, Chih-Ming Chen^3,4^, Tung-Yueh Lin^1^, Kai-Lun Liu^1^, Chien-Ming Wu^1^, Yun-Ching Chen^5^, Chih-Pin Chuu^6^, Ling-Yu Wang^7,8^, Hsing-Pang Hsieh^3,4,9^, Hsing-Jien Kung^10,11^, and Wen-Ching Wang^1*^

^1^Institute of Molecular and Cellular Biology and Department of Life Sciences, National Tsing-Hua University, Hsinchu City 30013, Taiwan.

^2^Department of Biotechnology and Pharmaceutical Technology, Yuanpei University of Medical Technology, Hsinchu City 30015, Taiwan.

^3^Institute of Biotechnology and Pharmaceutical Research, National Health Research Institutes, Zhunan Town, Maioli County 35053, Taiwan.

^4^Biomedical Translation Research Center, Academia Sinica, Taipei City 11571, Taiwan.

^5^Institute of Biomedical Engineering, National Tsing-Hua University, Hsinchu City 30013, Taiwan.

^6^Institute of Cellular and System Medicine, National Health Research Institutes, Zhunan Town, Miaoli County 35053, Taiwan.

^7^Graduate Institute of Biomedical Sciences, Division of Biochemistry, Molecular and Cellular Biology, Chang Gung University, Taoyuan City 33302, Taiwan.

^8^Division of Medical Oncology, Chang Gung Memorial Hospital, Linkou, Taoyuan City 33305, Taiwan.

^9^Department of Chemistry, National Tsing Hua University, Hsinchu City 30013, Taiwan.

^10^Department of Biochemistry and Molecular Medicine, University of California Davis School of Medicine, University of California Davis Cancer Centre, Sacramento, CA 95817.

^11^Graduate Institute of Cancer Biology and Drug Discovery, Taipei Medical University, Taipei City 11031, Taiwan.

*Correspondence: wcwang@gapp.nthu.edu.tw; Tel: +886-3-5742766

^†^Jai-Shin Liu and Wei-Kai Fang contributed equally to this work

^1^Institute of Molecular and Cellular Biology and Department of Life Sciences, National Tsing-Hua University, Hsinchu, 30013, Taiwan

**This file includes:**

**Figures S1 to S4**

**Tables S1 to S5**


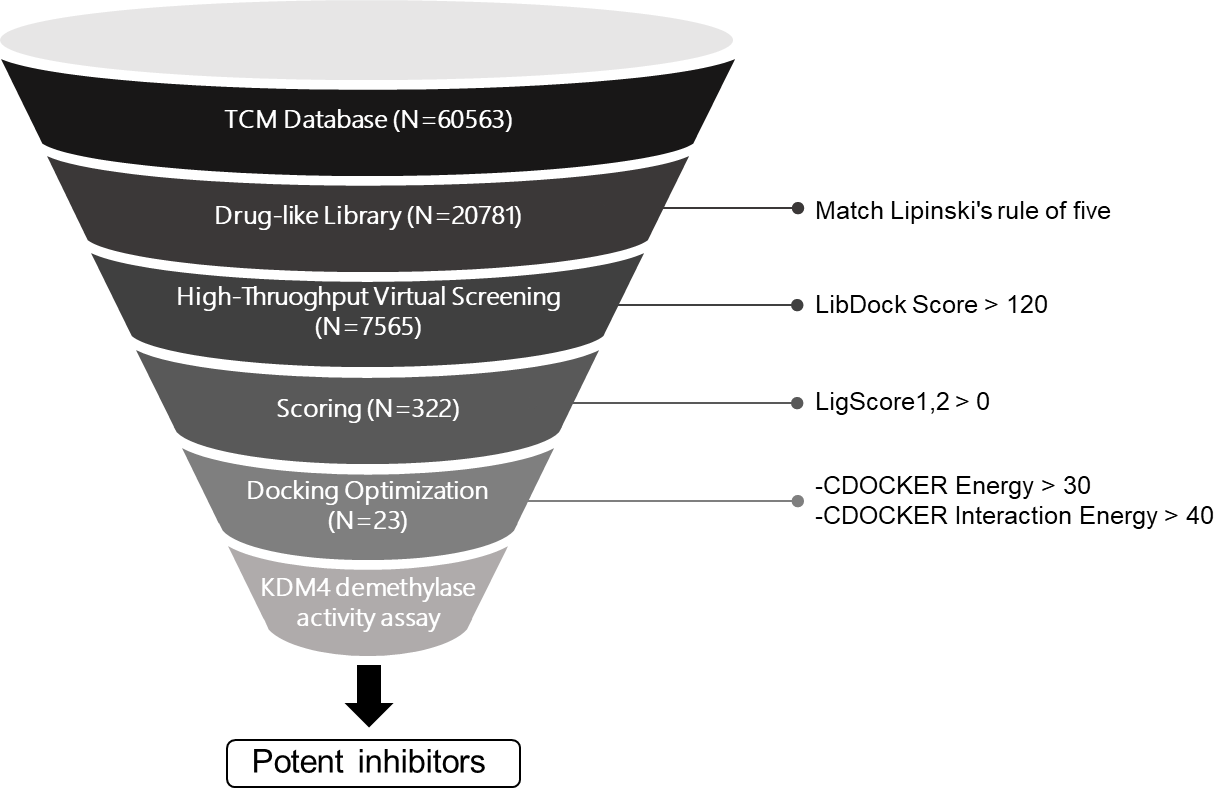


**Fig. S1** Schematic diagram of KDM4B inhibitor discovery from a library of natural products.


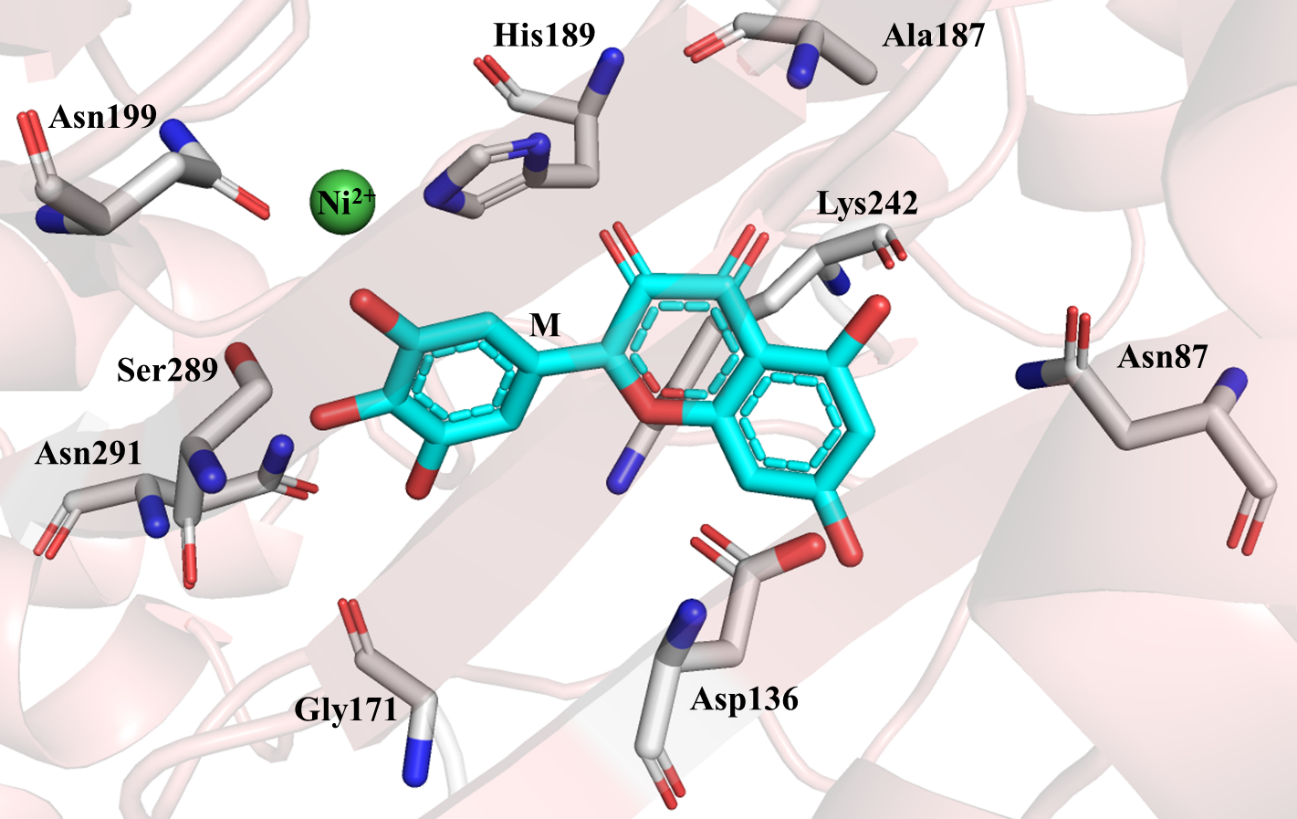


**A**

**B**


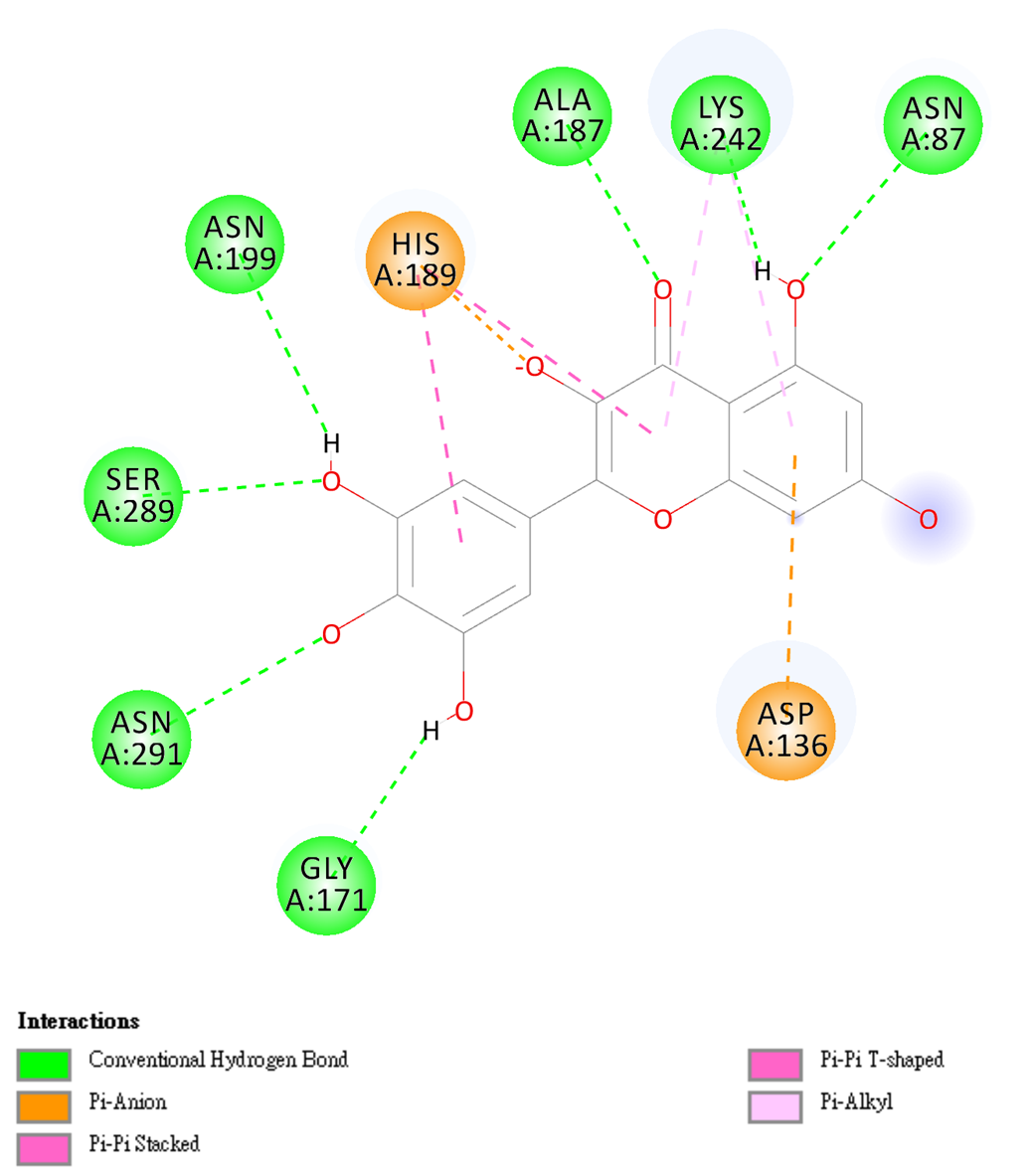


**Fig. S2** Analysis of interacting residues in the KDM4B-compound models. (A) The docked KDM4B·myricetin complex. Myricetin, M, occupied the catalytic pocket of KDM4B. (B) 2D diagram of KDM4B·myricetin interaction. Myricetin has seven H bonds, two Pi-anion, one Pi-Pi stacked, one Pi-Pi T-shaped, and one Pi-Alkyl interaction with KDM4B.


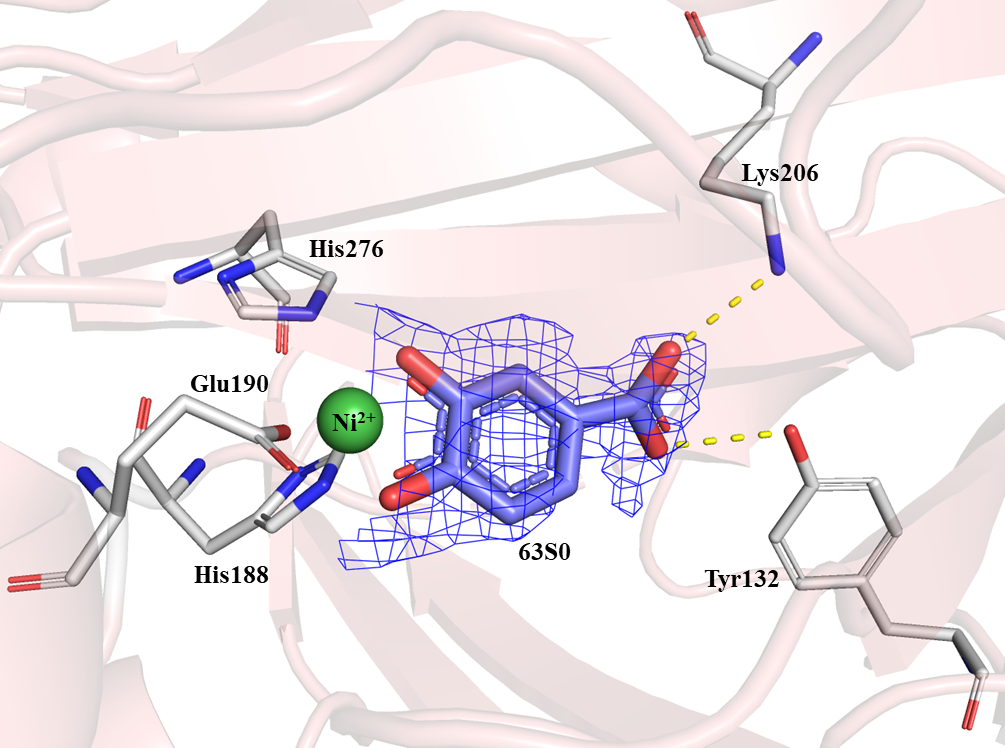


**Fig. S3** The $2F_{o}-F_{c}$ map of 63S0 in the liganded complex, contoured at 1.5 σ. The bound 63S0 is drawn as heavy blue sticks. The interacting residues are drawn in thin grey sticks. The oxygen and nitrogen atoms are colored in red and blue, respectively. The Nickel ion is drawn in green.


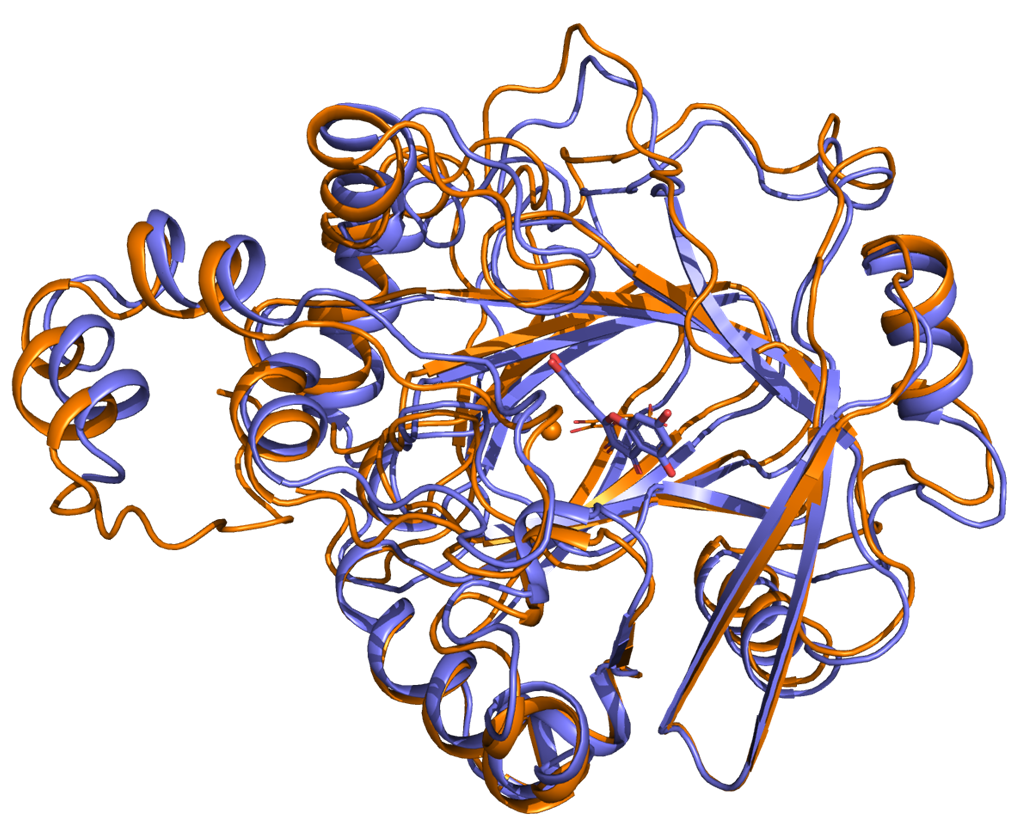


**B**

**A**


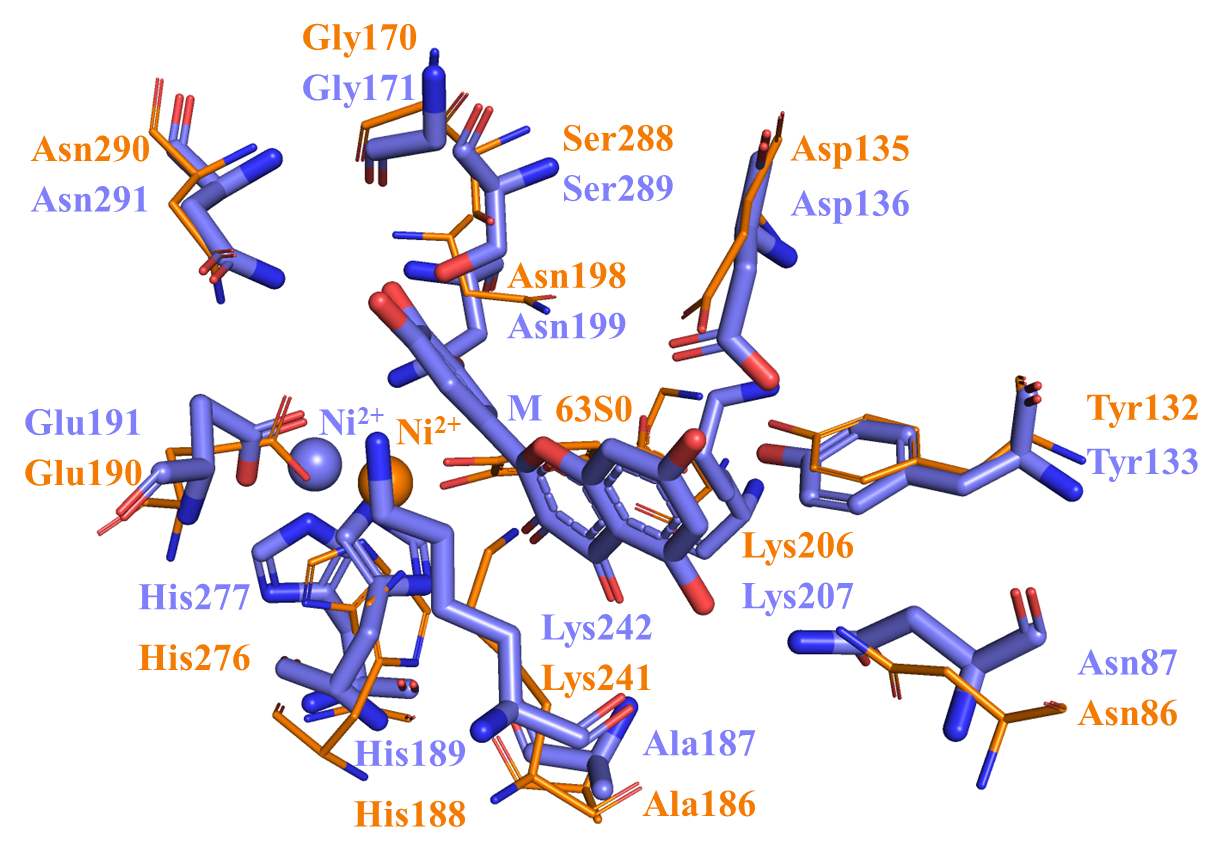


**Fig. S4** Superposition of KDM4A and KDM4B structures. (A) The KDM4A⋅63S0 crystal structure (orange) and the modeled KDM4B⋅myricetin structure (slate blue) are superimposed. (B) Superposition of active-site residues based on (A). The bound 63S0 and the nearby residues are shown as thin sticks. Myricetin and the surrounding residues are shown as thick sticks. The oxygen and nitrogen atoms are red and blue, respectively. KDM4A⋅63S0 and KDM4B⋅myricetin have the same β-jellyroll structural fold and highly similar active sites.

**Table S1** Inhibition effect of selected compounds from TCM database on KDM4B.

| **ID** | **Name** | **KDM4B Relative Activity (%)** |
| --- | --- | --- |
| Berberine | 5,6-dihydro-9,10-dimethoxybenzo[g]-1,3-benzodioxolo[5,6-a]quinolizinium | 31.1 |
| 33187 | 6-chloro-9-(tetrahydro-2H-pyran-2-yl)-9H-purine | 66.2 |
| 281654 | 1-(3,4,5-trimethoxyphenyl)-1-penten-3-one | 74.1 |
| 400588 | 2,2-diphenyl-1,3-benzodioxole | 71.6 |
| 107364 | 3-(3,4-dimethoxyphenyl)-1-(4-hydroxyphenyl)-2-phenyl-2-propen-1-one | 72.0 |
| 90931 | 1-(4-methoxyphenyl)-3-(3,4,5-trimethoxyphenyl)-2-propen-1-one | 49.0 |
| 643179 | 4,4-dimethyl-1-(3,4,5-trimethoxyphenyl)-1-penten-3-one | 64.5 |
| 21703 | 8-(1-piperidinyl)-9H-purin-6-amine | 61.3 |
| 11868 | 3-(3,4-dimethoxyphenyl)-1-phenyl-2-propen-1-one | 56.2 |
| 282168 | N-butyl-2,3,10-trimethoxy-13-methyl-5,6-dihydro-7 ^5^-isoquino[3,2-a]isoquinolin-9-amine | 54.9 |
| 191962 | 2,3,10,11-tetramethoxy-8-methyl-5,6-dihydro-7 ^5^-isoquino[3,2-a]isoquinoline | 60.0 |
| 282132 | N-benzyl-2,3,10-trimethoxy-13-methyl-5,6-dihydro-7 ^5^-isoquino[3,2-a]isoquinolin-9-amine | 32.6 |
| 282131 | N^1^-(2,3,10-trimethoxy-13-methyl-5,6-dihydro-7 ^5^-isoquino[3,2-a]isoquinolin-9-yl)-1,2-ethanediamine | 39.0 |
| Quercetin | 2-(3,4-dihydroxyphenyl)-3,5,7-trihydroxychromen-4-one | 5.3 |
| Kaempferol | 3,5,7-trihydroxy-2-(4-hydroxyphenyl)-4H-chromen-4-one | 13.0 |
| Myricetin | 3,5,7-trihydroxy-2-(3,4,5-trihydroxyphenyl)-4H-chromen-4-one | 2.9 |
| Catechins | (2R,3S)-2-(3,4-dihydroxyphenyl)-3,5,7-trihydroxychromen-4-one | 62.4 |
| Isohanetin | 3,5,7-trihydroxy-2-(4-hydroxy-3-methoxyphenyl)-4H-chromen-4-one | 41.1 |
| 3-Hydroxy-flavone | 3-Hydroxy-2-phenyl-4H-chromen-4-one | 52.4 |
| Galangin | 3,5,7-Trihydroxy-2-phenyl-4H-chromen-4-one | 45.0 |
| Gossypin | 2-(3,4-dihydroxyphenyl)-3,5,7-trihydroxy-8-[(2S,3R,4S,5S,6R)-3,4,5-trihydroxy-6-(hydroxymethyl)oxan-2-yl]oxychromen-4-one | 22.8 |
| Morin | 2-(2,4-dihydroxyphenyl)-3,5,7-trihydroxychromen-4-one | 36.9 |
| Rhamnetin | 2-(3,4-dihydroxyphenyl)-3,5-dihydroxy-7-methoxychromen-4-one | 71.9 |

**Table S2** Inhibition effects of analogues based on a myricetin’s fragment, (4-phenylpiperazine-1-yl) (phenyl) methanone, on KDM4A.

|  | | | | | | |
| --- | --- | --- | --- | --- | --- | --- |
| Compound | R_1_ | R_2_ | R_3_ | R_4_ | R_5_ | IC_50_ (μM)^a^ |
| BPRKD001S0 | - | OH | OH | OH |  | 5.03 |
| BPRKD002S0 | OH | OH | OH | - |  | 2.21 |
| BPRKD008S0 | - | - | OH | OH |  | 4.48 |
| BPRKD031S0 | - | - | OH | OH |  | 8.71 |
| BPRKD032S0 | - | - | OH | OH |  | 4.33 |
| BPRKD033S0 | - | - | OH | OH |  | 5.78 |
| BPRKD034S0 | - | - | OMe | OH |  | >10 |
| BPRKD035S0 | - | OH | OH | - |  | 5.60 |
| BPRKD041S0 | - | - | OH | OH |  | 4.27 |
| BPRKD042S0 | - | OH | COOH | - |  | >10 |
| BPRKD046S0 | - | - | OH | OH |  | 6.70 |
| ^a^ Values are means derived from three independent dose-response curves. KDM4A demethylase residual activity was determined by FDH-coupled assay as described in Methods. | | | | | | |

**Table S3** Inhibition effects of analogues based on a myricetin’s fragment, (E)-N'-(benzylidene) isonicotinohydrazide, on KDM4A.

|  | | | | | |
| --- | --- | --- | --- | --- | --- |
| Compound | R_1_ | R_2_ | R_3_ | R_4_ | IC_50_ (μM)^a^ |
| BPRKD007S0 | OH | OH | OH |  | 2.16 |
| BPRKD018S0 | OH | OH | OH |  | 1.26 |
| BPRKD022S0 | OH | OH | - |  | 0.86 |
| BPRKD023S0 | OH | OH | OH |  | 1.12 |
| BPRKD038S0 | - | OH | OH |  | 1.27 |
| BPRKD057S0 | OH | OH | - |  | 1.90 |
| BPRKD059S0 | OH | OH | - |  | 1.49 |
| ^a^ Values are means derived from three independent dose-response curves. KDM4A demethylase residual activity was determined by FDH-coupled assay as described in Methods. | | | | | |

**Table S4** Inhibition effects of analogues based on a myricetin’s fragment, pyrocatechol, on KDM4A.

|  | | | |
| --- | --- | --- | --- |
| Compound | R_1_ | R_2_ | IC_50_ (μM)^a^ |
| BPRKD020S0 | CHO | - | 2.79 |
| BPRKD026S0 | - | - | 3.14 |
| BPRKD027S0 | - | CHO | 1.26 |
| BPRKD029S0 | - |  | 0.92 |
| BPRKD063S0 | - | COOH | 0.56 |
| ^a^ Values are means derived from three independent dose-response curves. KDM4A demethylase residual activity was determined by FDH-coupled assay as described in Methods. | | | |

**Table S5** Crystallographic data and refinement statistics.

| **Structure** | KDM4A·63S0 |
| --- | --- |
| **Data collection** |  |
| Beamline | NSRRC BL-13B^a^ |
| Space group | P3_1_21 |
| Cell dimensions  a, b, c (Å)  α, β, γ (º) | 149.369, 149.369, 62.175  90.00, 90.00, 120.00 |
| Resolution (Å) | 30.0–2.60 |
| Highest resolution shell (Å) | 2.69–2.60 |
| Unique reflections | 24683 |
| Completeness (%)^b^ | 99.6 (96.1) |
| Average *I/σ* (*I*)^b^ | 43.7 (2.36) |
| *R_merge_* (%)^b,c^ | 7.0 (68.7) |
| Redundancy^b^ | 17.8 (12.0) |
| Solvent Content (%) | 50.34 |
| **Refinement** |  |
| Resolution range (Å) | 30.0–2.60 |
| Number of atoms | 2963 |
| Protein atoms | 2799 |
| Solvent atoms | 150 |
| Ligand atoms | 14 |
| *R_work_*^d^/*R_free_*^e^ | 16.8/22.2 |
| R.m.s.d. Bond lengths (Å)^f^ | 0.019 |
| R.m.s.d. Bond angles (º)^f^ | 1.959 |
| Overall *B*-factor (Å^2^) | 43.64 |
| Estimated coordinate error (Å) | 0.143 |
| **Ramachandran analysis (%)**^g^ |  |
| Preferred | 96.15 |
| Allowed | 3.85 |
| Outliers | 0.00 |
| ^a^ BL-13B beamline at National Synchrotron Radiation Research Center (NSRRC), Hsinchu, Taiwan.  ^b^ Values in parentheses refer to statistics in the highest-resolution shell.  ^c^ $R_{merge}=\sum\left\vert I_{obs}-\left\langle I \right\rangle\right\vert/\sum I_{obs}$.  ^d^ $R_{work}=\sum\left\vert F_{obs}-F_{calc} \right\vert/\sum F_{obs}$, where $F_{obs}$ and $F_{calc}$ are the observed and calculated structure-factor amplitudes, respectively.  ^e^ $R_{free}$ was computed using 5% of the data assigned randomly.  ^f^ Root mean square deviation.  ^g^ Estimated standard uncertainties based on maximum likelihood. | |
